# Brian2GeNN: accelerating spiking neural network simulations with graphics hardware

**DOI:** 10.1038/s41598-019-54957-7

**Published:** 2020-01-15

**Authors:** Marcel Stimberg, Dan F. M. Goodman, Thomas Nowotny

**Affiliations:** 1 Sorbonne Université, INSERM, CNRS, Institut de la Vision, Paris, France; 2 Department of Electrical and Electronic Engineering, Imperial College London, London, UK; 3 Centre for Computational Neuroscience and Robotics, Sussex Neuroscience, School of Engineering and Informatics, University of Sussex, Brighton, UK

**Keywords:** Computational neuroscience, Software

## Abstract

“Brian” is a popular Python-based simulator for spiking neural networks, commonly used in computational neuroscience. GeNN is a C++-based meta-compiler for accelerating spiking neural network simulations using consumer or high performance grade graphics processing units (GPUs). Here we introduce a new software package, Brian2GeNN, that connects the two systems so that users can make use of GeNN GPU acceleration when developing their models in Brian, without requiring any technical knowledge about GPUs, C++ or GeNN. The new Brian2GeNN software uses a pipeline of code generation to translate Brian scripts into C++ code that can be used as input to GeNN, and subsequently can be run on suitable NVIDIA GPU accelerators. From the user’s perspective, the entire pipeline is invoked by adding two simple lines to their Brian scripts. We have shown that using Brian2GeNN, two non-trivial models from the literature can run tens to hundreds of times faster than on CPU.

## Introduction

GPU acceleration emerged when creative academics discovered that modern graphics processing units (GPUs) could be used to execute general purpose algorithms, e.g. for neural network simulations^1,2^. The real revolution occurred when NVIDIA corporation embraced the idea of GPUs as general purpose computing accelerators and developed the CUDA application programming interface^3^ in 2006. Since then, GPU acceleration has become a major factor in high performance computing and has fueled much of the recent renaissance in artificial intelligence. One of the remaining challenges when using GPU acceleration is the high degree of insight into GPU computing architecture and careful optimizations needed in order to achieve good acceleration, in spite of the abstractions that CUDA offers. A number of simulators have used GPUs to accelerate spiking neural network simulations, but the majority do not allow for easily defining new models, relying instead on a fixed set of existing models^4–8^. Since 2010 we have been developing the GPU enhanced neuronal networks (GeNN) framework^9^ that uses code generation techniques^10,11^ to simplify the use of GPU accelerators for the simulation of spiking neural networks. GPUs, and in particular GeNN, have been shown to enable efficient simulations compared to CPUs and even compared to dedicated neuromorphic hardware^12^. Other simulators that have taken this code generation approach are Brian2CUDA^13^ (currently under development) and ANNarchy^14^ (Linux only).

Brian is a general purpose simulator for spiking neural networks written in Python, with the aim of simplifying the process of developing models^15–17^. Version 2 of Brian^18^ introduced a code generation framework^10,19^ to allow for higher performance than was possible in pure Python. The design separates the Brian front-end (written in Python) from the back-end computational engine (multiple possibilities in different languages, including C++), and allows for the development of third party packages to add new back-ends.

Here, we introduce the Brian2GeNN software interface we have developed to allow running Brian models on a GPU via GeNN. We analysed the performance for some typical models and find that–depending on the CPU and GPU used–performance can be tens to hundreds of times faster.

## Results

We benchmarked Brian2GeNN on two model networks that we named “COBAHH” and “Mbody”. COBAHH is an implementation of a benchmark network described by Brette *et al*.^20^ (for details, see Methods). Essentially, this benchmark model consists of *N* Hodgkin-Huxley-type neurons, modified from the model by Traub and Miles^21^, 80% of which form excitatory synapses and 20% inhibitory synapses. All neurons were connected to all other neurons randomly with a connection probability chosen such that each neuron received on average 1,000 connections for large models, or connections from all other neurons if the number of neurons was less than 1,000.

Mbody is an implementation of a previous model of the mushroom body^22^, but unlike in the original publication also with a similar neuron model to the one used for the COBAHH benchmark (for details, see Methods). The model was used with 100 projection neurons, 100 extrinsic Kenyon cells and varying numbers *N* of intrinsic Kenyon cells (hidden layer). Projection neurons in the input layer are connected with fixed probability of 15% to intrinsic Kenyon cells. Up to *N* = 10,000 intrinsic Kenyon cells are connected all-to-all to the extrinsic Kenyon cells, and for *N* > 10,000, they are connected randomly with probability chosen such that the extrinsic Kenyon cells receive input from on average 10,000 intrinsic Kenyon cells.

The COBAHH model is an example of a popular model type used for cortical microcircuits whereas the Mbody model is a typical feedforward network. COBAHH like models are used to investigate the dynamics of balanced networks and do not involve learning while the Mbody example is a prototypical model of a simple learning circuit, for instance for classification, and hence contains plastic synapses. The main audience for Brian 2 and GeNN are computational neuroscientists and we have therefore used models with conductance based neurons. The models were scaled so that the activity in the models was within sensible physiological limits, i.e. activity neither died out nor went into unrealistically high firing rates. The exact scaling and the details of the models are explained in the Methods.

Both models were integrated with an exponential Euler algorithm at 0.1 ms time steps. The benchmarks presented here were obtained using the GeNN sparse matrix representation for synaptic connections.

We benchmarked the models on different systems and with different backends. The GeNN backend through the Brian2GeNN interface presented here was compared to the “C++ standalone” backend included with the Brian simulator which runs on the CPU with either a single thread or with multiple threads via the OpenMP interface. Benchmarks were performed for both, single precision (32 bit) and double precision (64 bit) floating point. This is particularly relevant for GPUs because different GPU models have a different number of 64 bit cores, which in addition may be run at reduced clock frequencies for thermal management, and, therefore, can be between only 2× but up to 32× slower in double precision simulations than in single precision (see Table 1).

**Table 1:** Configurations used for benchmarking.

CPU			GPU				
# cores	Clock speed (GHz)	Memory (GB)	Architecture	# cores	Memory (GB)	Performance* (single)	Performance* (double)
**Intel Xeon E5-1630 v3**			**Quadro K2200**				
4	3.7-3.8	16	Maxwell	640	4	1,439	45
**Intel Xeon E5-1620 v2**			**Tesla K40c**				
4	3.7-3.9	32	Kepler	2,880	12	4,290	1,430
**Intel Core i9-7920X**			**TITAN Xp**				
12	2.9-4.4	64	Pascal	3,840	12	12,150	380
**Dual Intel Xeon Gold 6148**			**Tesla V100**				
2 × 20	2.4	192	Volta	5,120	16	14,131	7,066

*Maximum performance in GFLOPS.

We recorded the overall wall clock time for the simulation including all stages from code generation and initialization in Python to C++ compilation and execution of the binary (“overall runtime”). We also took more fine-grained measurements of the time for code generation and compilation, the time spent for synapse creation and initialization, the time spent for the actual simulation and the overhead, including, e.g., time spent on reformatting data structures between Brian 2 and GeNN formats, copying to and from the GPU and writing results to disk. All simulation times that we present here are the smallest out of three simulation runs with an identical setup.

We verified that the simulation results do not depend on the simulation method that was used (single- and multi-threaded simulation on the CPU via Brian; simulation on the GPU via Brian2GeNN and GeNN). However, the simulations that were performed for the benchmark results here cannot be directly compared with each other, since synaptic connections and variable initialisation are random. When we fix these connections and initialisations to be identical across runs, we do get highly reproducible simulation results: in a test recording all the spikes in a COBAHH network with 16,000 neurons over 10 s, all simulations with double precision gave exactly the same results, i.e. all spikes fell into identical time steps for all neurons. When using single precision, small numerical discrepancies (e.g. due to differences in the order of summations) added up and led to minor spike timing discrepancies between simulations on the CPU and the GPU. However, all neurons emitted the exact same number of spikes, the discrepancies were almost exclusively restricted to spikes occurring a single time step earlier or later; only 9 out of 16,000 neurons (i.e., 0.06%) had any spikes shifted by more than a single time step. For the Mbody benchmark, all simulations were completely identical across CPU- and GPU-based simulations in a test with 160,200 neurons over 1 s, both for single and double precision. Note that in general, numerical simulations performed on different platforms cannot be expected to always give results that are identical on a spike-by-spike basis, especially in recurrent networks. Validating simulation results across different technical approaches therefore requires comparing more global measures such as firing and correlation statistics^23^. A validation of this type has been performed previously for the GeNN simulator^12^.

### Simulation time

**Figure 1:**
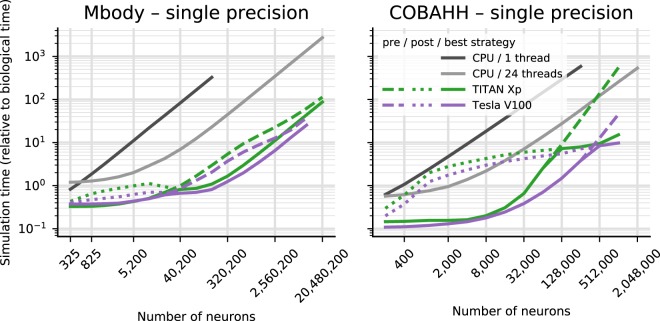
Benchmark of the net simulation time on a 12 core CPU with a single thread (dark gray) or using OpenMP with 24 threads (light gray), compared to a consumer GPU (TITAN Xp) and an HPC model (Tesla V100). For the GPUs, simulation times are displayed separately for a pre-synaptic parallelisation strategy (dotted) or post-synaptic strategy (dashed). The better of the two strategies is highlighted by a solid line.

The results for the net simulation time for the two models on CPU and the TITAN Xp and Tesla V100 GPUs are shown in Fig. 1 as a function of the size of the models, indicated by the total number of neurons. GeNN offers two different strategies for parallelising the spike propagation logic, along pre-synaptic inputs (looping over post-synaptic targets) or along post-synaptic targets (looping over pre-synaptic sources). We benchmarked both algorithms for each of the models.

The single thread CPU solution scales essentially linearly with the size of the two models, expressed in terms of the number of simulated neurons (Fig. 1). This reflects the linear scaling of processing time with the number of operations required and that both models are essentially neuron-bound on the CPU due to their computationally expensive neuron model, their chosen connectivity and the observed number of spikes. The 24-thread OpenMP simulations take initially the same time for very small models but we found that the simulations ran about 13–14 times faster than on a single CPU core for the largest MBody model tested on a single CPU core (160,200 neurons) and 8–11 times faster for the largest COBAHH model tested on a single CPU core (256,000 neurons). Larger models were only tested on 24-thread OpenMP and GPUs due to the prohibitively long runtime on a single CPU core. For models larger than 40,200 neurons (Mbody) and 8,000 neurons (COBAHH), the 24 thread OpenMP solution also scales approximately linearly with the number of neurons.

The simulations run on the GPU via Brian2GeNN (green and purple lines in Fig. 1) were significantly faster than the 24 thread OpenMP (light gray), for instance, 40–54 times faster in the Mbody model for 10,240,200 neurons and up to 24–26 times faster in the COBAHH model for 1,024,000 neurons when using the Tesla V100 GPU. We have summarised the observed speed-ups achieved for the simulation time in Table 2

**Table 2:** Speed-up on GPUs.

Mbody benchmark												
	*compared to CPU 1 thread*						*compared to CPU 24 thread*					
**# neurons**	40,200		80,200		160,200		40,200		160,200		10,240,200	
Quadro K2200	*39.2*	6.5	*52.0*	6.7	*60.7*	7.0	*3.3*	0.6	*4.3*	0.5	*4.3*	0.5
Tesla K40c	*34.7*	25.6	*58.8*	39.8	*80.9*	53.8	*2.9*	2.4	*5.7*	4.2	*7.1*	5.8
Titan Xp	*101.9*	39.4	*190.3*	51.4	*300.3*	60.9	*8.5*	3.7	*21.1*	4.8	*31.0*	5.0
Tesla V100	*124.3*	105.9	*235.5*	191.7	*401.6*	251.7	10.4	9.9	28.3	19.7	*53.9*	40.4
**COBAHH benchmark**												
**# neurons**	64,000		128,000		256,000		64,000		256,000		10,24,000	
Quadro K2200	*18.0*	4.9	*24.2*	4.5	*13.6*	4.1	*1.7*	0.6	*1.3*	0.5	–	–
Tesla K40c	*20.6*	16.3	*11.7*	9.4	*16.7*	8.6	*1.9*	1.9	*1.6*	1.1	*1.0*	–
Titan Xp	*57.7*	29.1	*40.3*	22.2	*73.8*	33.7	*5.4*	3.4	*7.2*	4.1	*16.9*	6.5
Tesla V100	*207.7*	155.7	*196.5*	134.4	*154.1*	113.5	*19.6*	18.3	*15.0*	13.9	*26.3*	24.2

This only considers simulation time. Numbers are relative to simulations on the host of the Titan Xp GPU (see Table 1) and compare to a single-thread simulation (left) or a 24-thread OpenMP simulation (right). The two numbers shown are for single precision (italic) and double precision (normal font). The underlined numbers are the highest and lowest observed speed-ups for each of the four quadrants of the table.

. Overall the GPU runs always faster than a single threaded CPU version, up to a factor of 400, but when compared against the 24 thread OpenMP version, acceleration can vary from 2× slower than the CPU to about 50× faster.

Interestingly, the different parallelisation methods for spike propagation available in GeNN (dashed and dotted lines in Fig. 1) perform differently as a function of size. The post-synaptic method is always faster for small models while the pre-synaptic method wins for very large models. This trend is the same for both tested models, but the exact crossover point where pre-synaptic parallelisation becomes more efficient depends on the model, and to a lesser degree on the GPU hardware. For the Mbody example, the swap occurs at moderate model sizes of about 40200 neurons, whereas for the COBAHH model, it is for much larger models (128,000 neurons for the TITAN Xp and 512,000 neurons for the Tesla V100). Also, while the differences of the two methods are not that pronounced for the large Mbody models, the post-synaptic method in the COBAHH model scales very poorly with size at large model sizes, leading to quite low performance of Brian2GeNN in this mode. The pre-synaptic method, on the contrary, is not particularly fast for smaller to medium sized COBAHH models (even slower than the 24 thread OpenMP version), but scales excellently for the largest models, leading to significant speedups over OpenMP.

The general trend of the post-synaptic method being faster for small models and the pre-synaptic method for large models can be understood based on how these methods work and based on the scaling method of the benchmark models. In the post-synaptic parallelisation method, each post-synaptic neuron occupies its own thread and there is a loop over incoming spikes. This is efficient when there are many post-synaptic neurons and few incoming spikes. In contrast, in the pre-synaptic method, each emitted spike is processed in a separate thread, and there is a loop over the affected post-synaptic neurons. This method is better if there are many spikes and few or a moderate number of post-synaptic targets. In both models, the number of post-synaptic targets during scaling is constant (or capped for the iKCeKC synapses), but the number of spikes grows with the size of the model. In small models, there are few spikes and relative to the small spike number many post-synaptic targets - the post-synaptic method is better. For much larger models, there are many more spikes but roughly the same number of post-synaptic targets for each spike, so the pre-synaptic method becomes better. When, however, the exact crossover between the methods occurs, is hard to predict and can also depend on the GPU and the details of how the connectivity and the activity in the models scale.

**Figure 2:**
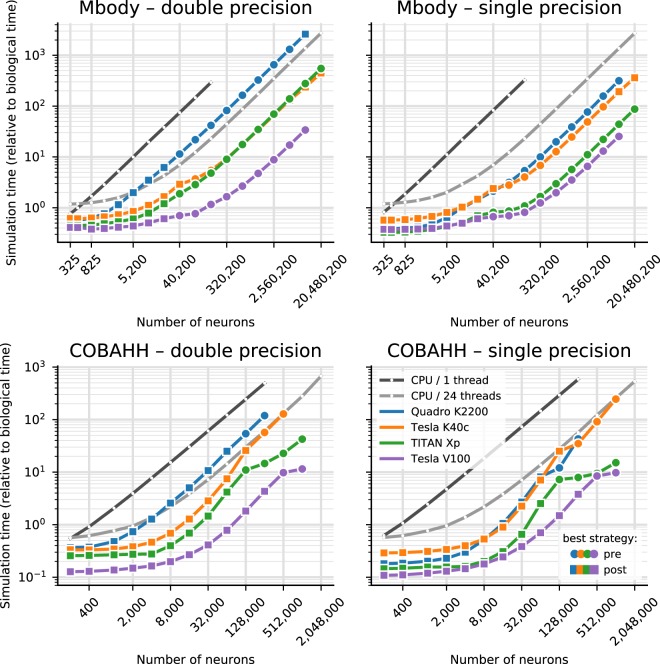
Benchmarking of the net simulation time for different GPU models. Measurements were taken separately for the MBody model (top) and COBAHH model (bottom) for double precision floating point (left) and single precision (right). Simulation time is shown relative to the simulated biological time (10 s). CPU performance was measured on the host of the TITAN Xp GPU (see Table 1). For the GPUs, the better (smaller) of the simulation times for either pre-synaptic or post-synaptic parallelisation strategy are shown; circles mark the simulations where the pre-synaptic strategy was faster, and squares those where the post-synaptic strategy was faster. See Fig. 1 and main text for more in-depth explanation.

The simulation times for a larger variety of different GPU hardwares are shown in Fig. 2. Note that we here display the results for the better of the two parallelisation strategies for each model run. We benchmarked four different graphics cards (see Table 1). The results show consistent trends given the specifications of the hardware (Table 1), even though some may not be as obvious as others. The Tesla V100 is almost always fastest, typically followed by the TITAN Xp, Tesla K40c and Quadro K2200 card in this order. Note however, the marked difference in double precision performance for the consumer cards (Quadro K2200 and TITAN Xp), compared to the high performance computing cards (Tesla K40c and Tesla V100): In Fig. 2, the blue and green lines are at markedly higher values on the left plots than on the right, while the orange and purple lines barely change between single and double precision plots. This is expected because the consumer cards have NVIDIA GPU architectures (Maxwell respectively Pascal) that have fewer double precision cores and double precision operations are hence up to 32 times slower than single precision, while the HPC cards used here are Kepler and Volta architecture and have only a factor 2 performance difference between double precision and single precision operations. Accordingly, while in single precision, the presumably less powerful but more recent Quadro K2200 card performs at the level of or even better than the older but larger Tesla K40c accelerator, it does not compare favourably for double precision.

Comparing the two models, it is clear that the performance gains of Brian2GeNN on the different GPU platforms is more marked for the Mbody model than for the COBAHH model. This would be expected for the spike propagation code because the mainly feedforward structure of the Mbody model lends itself better to parallelisation on GPUs with GeNN than the randomly recurrently connected COBAHH model. It should be noted that spike propagation is the most challenging aspect of running neural network simulations on GPUs^24^, and it typically takes up a larger share of the total computation time compared to simulations on CPUs. We can see this pattern in the examples presented here (executed on the Intel Core i9-7920X CPU with a Titan Xp GPU): in a single precision simulation of the COBAHH model with 512,000 neurons, the synaptic propagation takes up 47% of the time when run on the CPU (24threads), but 82% when run on the GPU; in a simulation of the Mbody model with 10,240,200 neurons, synaptic propagation, including updates of the plastic synapses, takes up only 1–2% of the time on the CPU but 20% on the GPU.

### Time for other tasks

**Figure 3:**
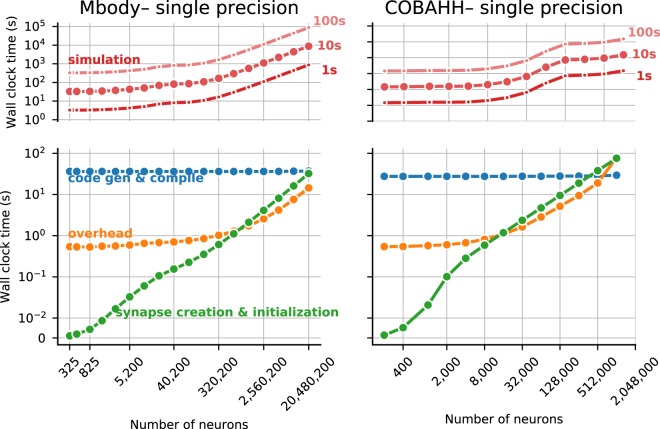
Overview of the components that make up the total runtime of a simulation for the Mbody (left) and the COBAHH benchmark (right). The top panels show the time spent in the simulation itself which scales with the biological runtime of the model (shown at the right) and dominates the overall runtime for big networks and/or long simulations. Simulation times were measured for biological runtimes of 10 s (middle line), while the times for runs of 1 s (bottom line) and 100 s (top line) were extrapolated. The bottom panels show the time spent for code generation and compilation (blue), general overhead such as copying data between the CPU and the GPU (orange), and the time for synapse creation and the initialization of state variables before the start of the simulation (green). The details shown here are for single-precision simulations run on the Titan Xp GPU.

So far we have presented results for the core simulation time. As explained in the methods, Brian2GeNN has a substantial pipeline of tasks before and after the main simulation takes place. Figure 3 illustrates the essence of how the computation times along this pipeline stack up. We defined four main phases of a Brian2GeNN run: “code generation and compilation”, “synapse creation”, “main simulation” and “overheads”, which bundles smaller tasks such as transforming data formats between Brian 2 format and GeNN format, copying from and to the GPU and writing results to disk. For illustration we have used the data from the TITAN Xp card and Intel Core i9-7920X CPU. The data in the top two panels in Fig. 3 repeats the results for the simulation time but also shows extrapolations for shorter and longer runs, where computation times are strictly proportional to the length of simulated biological time. This phase is almost entirely determined by the GPU model. The bottom two panels show the compute time spent on the other three phases, which are determined by the CPU and the CPU-GPU bandwidth (for copying data to the GPU). Code generation and compilation is a fixed cost that is completely independent of the model size. On the contrary, computation time for synapse creation and initialisation increases linearly with model size in terms of the number of neurons. The other overheads are initially almost independent of model size but then also start increasing with the number of neurons. In the balance, for small to mid-sized models and short simulation runs (1 s biological time), code generation and compilation dominates the overall runtime whereas for large models and longer runs, the time for the main simulation dominates.

**Figure 4:**
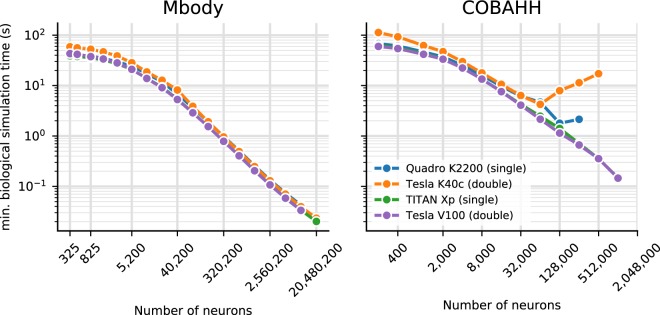
Minimal biological runtime after which the total simulation time, including preparations such as code generation and compilation (cf. Fig. 3), is smaller when using a GPU compared to 24 threads on a CPU, for networks of different sizes. This data was calculated from benchmark results as displayed in Fig. 2. The CPU comparison is the host of the Titan Xp GPU (see Table 1). Results for the Mbody benchmark (left) and the COBAHH benchmark (right). The calculations are based on single precision performance for the Quadro GPU (blue) and Titan Xp GPU (green), and on double precision performance for the Tesla K40c (orange) and the Tesla V100 GPU (purple).

To give a rough guide at which amount of biological time for any given model size it becomes viable to use Brian2GeNN we have calculated the minimum simulated biological time for which the overall runtime for Brian2GeNN is smaller than a 24 thread OpenMP solution (Fig. 4). For simulated biological time of 100 s or more it is always faster to use Brian2GeNN, regardless of model size or employed GPU accelerator. For shorter simulated time it depends on the simulated model and the GPU. For example, simulating 10 s biological time is perfectly viable on a Tesla V100 for the Mbody model at size 40,200 but would be slower on a Tesla K40c; or, simulating 10 s biological time would not be viable for any of the tested GPUs for the COBAHH model at size 8,000 but viable on all of them at size 64,000.

## Discussion

In designing software for computational neuroscience, there are two seemingly conflicting requirements: for high performance and for flexibility. The ability to create new types of models is essential for research that goes beyond what is already known at the time that a simulator package is created. However, hand written code that implements particular models can be much more computationally efficient. This is particularly true in the case of GPU simulations, as it is difficult to make maximally efficient use of GPU resources. Consequently, almost all GPU-based simulators for spiking neural networks have not made it possible to easily create new user-defined neuron models^4–8^. The exceptions are GeNN, the package Brian2CUDA^13^ currently under development, and ANNarchy^14^, which is discussed below.

The technique of code generation allows us to solve this apparent conflict, and has been used by both the GeNN and Brian simulators^9,10,19^ as well as a number of other neural simulators^11^. In the case of GeNN, when writing a new model users need to write only a very small section of generic C++ code that defines how the variables of a neuron model are updated, and this is then inserted into a detailed template that allows that model to be simulated efficiently on a GPU. Brian meanwhile allows users to write their model definition at an even higher level, as standard mathematical equations in a Python script. These are then automatically converted into low-level C++ code to be compiled and executed on a CPU. In both cases, users write high level code (short snippets of C++ in the case of GeNN, or Python/mathematics in the case of Brian) and efficient low level code is automatically generated.

Linking Brian and GeNN accomplishes two tasks. Firstly, it allows existing Brian users to make use of a GPU to run their simulations without any technical knowledge of GPUs (via GeNN). Secondly, it gives GeNN users a high level and feature packed interface (Brian and Python) to manage their simulations. GeNN was originally designed to be used at the C++ level, with network setup and simulation management handled by the user in C++, but not all computational neuroscientists are comfortable working at this level and there can be considerable savings in development time working at a higher level.

The only other spiking neural network simulation package to allow for flexible model definition in a high level language, and for code to run on GPUs, is ANNarchy^14^. This simulator was originally designed to adapt a model definition syntax similar to Brian’s to rate-coded networks (rather than spiking neural networks), and to make use of GPUs for high performance. It has subsequently been updated to allow for the definition of spiking neural networks as well as hybrid networks, and simulating spiking networks on the GPU is now provided as an experimental feature. In contrast to Brian2GeNN which supports all major operating systems, ANNarchy only supports running simulations on the GPU on Linux.

As noted in^24^, on GPUs it is unlikely that there is a single best algorithm for spiking neural network simulation, but rather the best algorithm will depend on the model. A diversity of GPU spiking neural network simulator packages is therefore desirable.

Brian’s framework for defining models of neurons, synapses, networks and computational experiments is designed to be as expressive and flexible as possible. Consequently, not all features of Brian are available in GeNN, and not all simulations that can be run in GeNN will run efficiently. Among the most important currently unsupported features are continuous, i.e. not spike-based, connections (used for example to implement electrical synapses); heterogeneous, i.e. synapse-specific, synaptic delays; arbitrary, time-varying continuous stimuli; and complex simulation schedules (for example, multiple simulation runs or different simulation time steps for individual groups of neurons/synapses). Attempting to use an unsupported Brian feature with Brian2GeNN will simply raise an error.

However, some features that are supported may also lead to slow code on the GPU. This is because efficient use of the GPU requires appropriate paralellisation strategies and specific memory access patterns, and for some features (particularly relating to backpropagation of information in synapses) it is very difficult to arrange data in memory so that it can be accessed efficiently for both, forward and backward propagation on the GPU^24^. The very different scaling of runtimes in the COBAHH example for pre- and post-synaptic parallelisation strategies for synaptic updates in large model instances, as seen in Fig. 1, is a very typical example of such phenomena. However, it is not straightforward to predict when problems of this kind will be significant. The Mbody example has STDP but because it is otherwise well suited for GeNN due to essentially feedforward connectivity for the majority of synapses and sparse firing, it speeds up well in Brian2GeNN. The COBAHH example does not have plasticity and yet, due to its relatively dense, random connectivity and somewhat higher firing rates, the speedups are good but less pronounced than in the Mbody example. Ideally, one would like to be able to predict the likelihood and magnitude of an expected speedup for a given model on a given GPU but this is a notoriously difficult problem^24^. We therefore encourage users to simply try Brian2GeNN on their script, which can be done by adding just two lines to their script importing brian2genn and selecting the ‘genn’ device.

A general limitation of running simulations on GPU rather than CPU is memory, as GPUs typically have much less available memory. At the time of writing, the largest memory available on a GPU is 32GB on the extremely expensive V100, while consumer cards have less than 12GB. Available RAM typically limits maximum simulation size due to synaptic weight matrices (very close to 8 bytes per synapse for single precision or 12 bytes per synapse for double precision). For example, the COBAHH simulation at the largest sizes has around 60–70 times as many synapses as the Mbody simulation, meaning that maximum simulation sizes (as measured by the number of neurons) for the Mbody simulation are larger than for COBAHH (Fig. 2).

Further work on Brian and GeNN will go in two main directions. On the GeNN side, we plan to expand the features available in GeNN to cover more of the features available in Brian, as well as improving efficiency. A specific bottleneck that has been recently identified is the synapse creation task (see Fig. 3). Work is under way that enables synapse creation on the GPU instead of the CPU with considerable performance advantages, in particular where synaptic connectivity becomes more intricate.

On the Brian side, we plan to simplify and optimise the process of writing third party back-ends. This will not only simplify future development of Brian2GeNN but will also encourage the development of an ecosystem of back-ends, for example featuring different GPU algorithms or targeting different computational hardware such as field programmable gate arrays (FPGAs). An interface to generate CUDA code directly from a Brian script, called Brian2CUDA^13^, is also under development, but has not yet been released. Note that Brian2CUDA uses different data structures and algorithms than GeNN, and Brian, Brian2CUDA and GeNN are all developed by independent teams, and it is therefore likely that both GeNN and Brian2CUDA will be useful for different modelling requirements.

For Brian2GeNN itself, we are planning to expose more of the optimisation choices offered to direct users of GeNN to Brian2GeNN users, for instance per-synapse group choices for connectivity matrix representations (sparse, dense, ragged, bitmask) and parallelisation strategies (pre- or post-synaptic). We will also work on exposing the emerging on-GPU initialisation methods mentioned above and the heterogeneous synaptic delays that were recently introduced to GeNN.

## Methods

### Brian2GeNN

Brian2GeNN makes use of the existing code generation facilities in the Brian and GeNN simulators. These code generation facilities differ in important aspects. The Brian simulator provides a comprehensive code generation framework that converts not only high-level descriptions of neural and synaptic models to executable code, but also extends this framework to model initialization including the generation of synapses according to high-level rules. In addition, the user code is written in Python, a language that is very accessible to researchers with a less technical background. However, the generated code is C++ code that runs only on the CPU, and therefore cannot make use of the computational power of GPU accelerators. GeNN’s code generation framework on the other hand is focused more on organizing the code to run efficiently on highly parallel GPUs, leaving the task of defining the code for simulating the neural and synaptic model, and the details of how to run the overall simulation to the user. This is completed in C++, which allows tight integration with other C++ based code, e.g. in the context of robotic controllers, but also makes writing a GeNN simulation relatively difficult for inexperienced programmers. The major advantage of using GeNN is its ability to generate efficient CUDA code that can be executed on a GPU to accelerate simulations.

**Figure 5:**
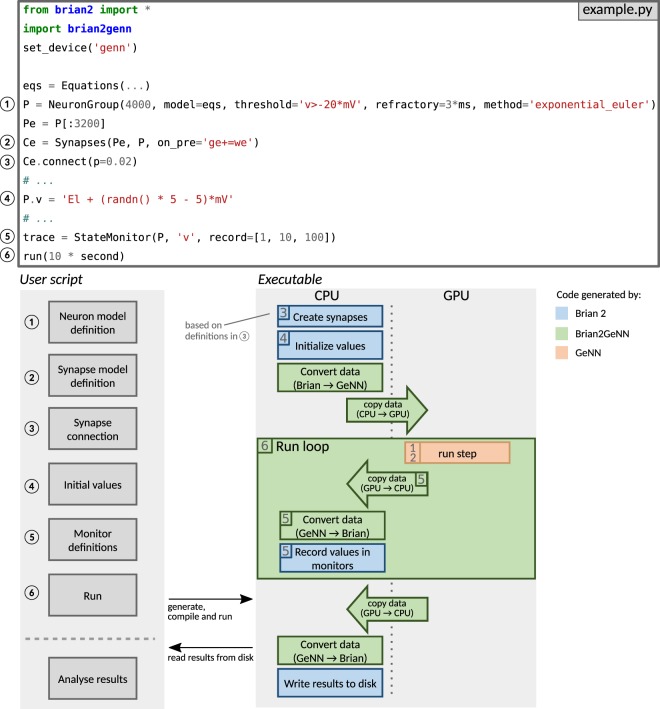
Running simulations with Brian2GeNN. Top: Excerpt from an example Brian script that will execute in a hybrid Brian/GeNN simulation due to the import of the brian2genn library (line 2) and the selection of the “GeNN device” (line 3). Bottom left: Typical workflow of a Brian2GeNN simulation: the run call triggers the code generation, compilation and execution. After the successful run, results are stored to disk and made available to the Python script. Numbers refer to the corresponding lines in the example code on top. Bottom right: Structure of generated code. Parts of the code result from Brian’s standard code generation process (blue), while the main run step is implemented by GeNN (red) and everything is connected together by Brian2GeNN (green). The preparation of the simulation and actions such as variable monitoring are executed on the CPU (left), while the core of the simulation is executed on the GPU (right). The numbers in the boxes refer to the elements of the example code (top) and general schematic (bottom left) which are the base for the code of the corresponding block. For example, the “run step”, i.e. the advancement of the state variables of neurons and synapses at every time step, is based on the definitions of the neuron and synapse models in (1) and (2).

Brian2GeNN acts as a “glue” between Brian and GeNN, thereby combining the advantages of both simulators. It is built as an extension of Brian’s code generation mechanism and can therefore be directly used from within a Brian script; by choosing the “GeNN device” (lines 2–3, Fig. 5 top), a standard Brian simulation is turned into a hybrid Brian/GeNN simulation. Such a script typically sets up the simulation components and then triggers the simulation of the network (Fig. 5 top and bottom left). At this point, the code generation process is activated and generates, compiles and executes the target code. The results of this simulation are then written to disk by the executed code, enabling the Python code to access the requested results to analyze or plot them. The executable code (Fig. 5 bottom right) is jointly generated by Brian (blue boxes), Brian2GeNN (green boxes/arrows), and GeNN (red box) and executed partly on the CPU and partly on the GPU. The initial steps, synapse creation and model initialization, are unchanged from Brian’s default code generation process. However, since Brian and GeNN use different data structures to represent synapses, Brian2GeNN has to generate code to convert between the two formats. In addition, it copies all the data to the GPU so that it can be used during the simulation run. The main simulation loop delegates the core of the simulation, the dynamic update of neural and synaptic state variables as well as the propagation of synaptic events, to the code generated by the GeNN simulator, which executes on the GPU. After each time step, some of this data may be copied back from the GPU and converted to the Brian format so that it can be recorded by Brian’s monitoring mechanism. After the end of the simulation run, Brian2GeNN takes care to copy all data back from the GPU and to convert it to the Brian format, so that Brian can store the results to disk and make them available for analysis in the Python script.

### Benchmark models

We benchmarked Brian2GeNN on two models, named COBAHH and Mbody.

#### COBAHH model

**Figure 6:**
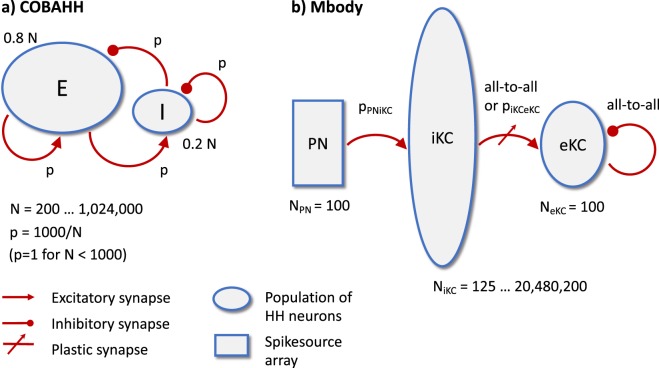
Diagrams of the two benchmark models, COBAHH (**a**) and Mbody (**b**). The COBAHH model is fully recurrent, whereas the Mbody model is essentially feedforward with exception of some all-to-all inhibition among the 100 eKCs.

The COBAHH model is a model frequently used in simulations of cortical structures and consists of two populations (Fig. 6a), a population of 0.8·*N* excitatory neurons and 0.2·*N* inhibitory neurons, where *N* denotes the total number of neurons, which was scaled from 200 to 1,024,000. Each pair of neurons is connected by a synapse with fixed probability *p* = 1,000/*N* (i.e., pairwise Bernoulli-distributed, potentially including self-connections), so that on average each neuron receives 1,000 inputs, regardless of scaling. For *N* < 1,000, *p* is set to 1, i.e. the network is all-to-all connected. Synapses are modeled as conductance based synapses,

1 $${I}_{{\rm{syn}}}={g}_{E}({V}_{E}-{V}_{{\rm{post}}})+{g}_{I}({V}_{I}-{V}_{{\rm{post}}})$$

2 $$\frac{d{g}_{E}}{dt}=-\frac{{g}_{E}}{{\tau }_{E}}+{w}_{E}\sum _{i}\,\delta (t-{t}_{i})$$

3 $$\frac{d{g}_{I}}{dt}=-\frac{{g}_{I}}{{\tau }_{I}}+{w}_{I}\sum _{i}\,\delta (t-{t}_{i})$$

where *g*_*E*_ is the conductance of the synapse at time *t*, *w*_*E*_ is the “weight” of the synapse, *τ*_*E*_ = 5 ms is the timescale of synaptic PSCs, *V*_*E*_ = 0 mV is the reversal potential and *t*_*i*_ denotes the spike times of the pre-synaptic neuron. The sum is over all pre-synaptic spikes and *δ* represents the Dirac *δ* distribution. The symbols are analogous for the inhibitory synapses with values *τ*_*I*_ = 10 ms and *V*_*I*_ = −80 mV. The weights for synapses were chosen as *w*_*E*_,*w*_*I*_ = *ω*·10^−9^ nS, where $\omega  \sim U\mathrm{([0,1])}$ is a uniformly distributed random variable on the interval [0,1] and the synaptic conductances were initialized independently according to the following normal distributions: ${g}_{E} \sim N\mathrm{(40}\,{\rm{nS}}\mathrm{,(15}\,{\rm{nS}}\,{)}^{2})$ and ${g}_{I} \sim N\mathrm{(200}\,{\rm{nS}}\,,\,{\mathrm{(120}{\rm{nS}})}^{2})$. Note that there is no substantial effect of the recurrent synapses due to the very low weight values *w*_*E*_ and *w*_*I*_. This has been done on purpose, so that scaling the network does not affect the network activity. However, since the values are non-zero, the simulations still include all the computations to propagate the synaptic activity and are therefore representative for benchmarking purposes.

Neurons were modeled by Hodgkin-Huxley type conductance based model equations,

4 $${C}_{M}\frac{dV}{dt}={g}_{L}({V}_{L}-V)+{g}_{{\rm{Na}}}{m}^{3}h({V}_{{\rm{Na}}}-V)+{g}_{{\rm{K}}}{n}^{4}({V}_{{\rm{K}}}-V)+{I}_{{\rm{syn}}}$$

where *C*_*M*_ = 0.2 nF is the membrane capacitance, *g*_*L*_ = 10 nS, *g*_Na_ = 20 μS, *g*_K_ = 6 μS are the maximal conductances, *V*_*L*_ = −60 mV, *V*_Na_ = 50 mV and *V*_K_ = −90 mV the reversal potentials and the activation variables have dynamic equations of the form

5 $$\frac{dx}{dt}={\alpha }_{x}\mathrm{(1}-x)-{\beta }_{x}x$$

where *x* = *m*, *h*, or *n*. The rate curves *α*_*x*_ and *β*_*x*_ are summarised in Table 3.

**Table 3:** Activation and inactivation rate curves as functions of *v* = *V*/[mV].

Variable	*α* [kHz]	*β* [kHz]
*m*	$\displaystyle {\alpha }_{m}=0.32\frac{-50-v}{{e}^{\frac{-50-v}{4}}-1}$	$\displaystyle {\beta }_{m}=0.28\frac{23+v}{{e}^{\frac{23+v}{5}}-1}$
*h*	$\displaystyle {\alpha }_{h}=0.128{e}^{\frac{-46-v}{18}}$	$\displaystyle {\beta }_{h}=\frac{4}{1+{e}^{\frac{-23-v}{5}}}$
*n*	$\displaystyle {\alpha }_{n}=0.032\frac{-48-v}{{e}^{\frac{-48-v}{5}}-1}$	$\displaystyle {\beta }_{n}=0.5{e}^{\frac{-53-v}{40}}$

Membrane potentials were initialized independently as $V\mathrm{(0)} \sim N({V}_{L}-5\,{\rm{mV}},{\mathrm{(5}{\rm{mV}})}^{2})$. Spikes were detected whenever the membrane potential *V* surpassed *V*_thresh_ = −20 mV and neurons were refractory, i.e. could not produce further spikes, for 3 ms after each spike.

#### Mbody model

The Mbody model is essentially a feedforward network inspired by the mushroom body of insects. As illustrated in Fig. 6b, there are three neuron populations, the projection neurons (PNs) of the antennal lobe, the so-called intrinsic Keynon cells (iKCs) of the mushroom body calyx, and the extrinsic Kenyon cells (eKCs) of the mushroom body lobes. PNs project to iKCs with a random connectivity, where each synapse exists with probability *p*_PNiKC_ = 0.15 (i.e., pairwise Bernoulli-distributed). For networks with less than 10,000 iKCs, *N*_iKC_ ≤ 10,000, the connections between iKCs and eKCs are all-to-all. For *N*_iKC_ > 10,000 they are random with fixed probability *p*_iKCeKC_ = 10,000/*N*_iKC_ for each connection to exist. This will on average connect 10,000 iKCs to each eKC. In addition to the feedforward connections, eKCs inhibit each other laterally with an all-to-all connectivity (including self-connections). Synapses are described as conductance based synapses,

6 $${I}_{{\rm{syn}}}={g}_{x}({V}_{x}-{V}_{{\rm{post}}})$$

7 $$\frac{d{g}_{x}}{dt}=-\frac{{g}_{x}}{{\tau }_{x}}+{w}_{x}\sum _{i}\,\delta (t-{t}_{i})$$

where *g*_*x*_ are the time dependent conductances of the synapses, *w*_*x*_ stands for the synapse weights, *V*_*x*_ stands for *V*_PNiKC_ = *V*_iKCeKC_ = 0 mV and *V*_eKCeKC_ = −92 mV, and *τ*_*x*_ for the synaptic timescales of *τ*_PNiKC_ = 2 ms, *τ*_iKCeKC_ = 10 ms, and *τ*_eKCeKC_ = 5 ms, respectively. The sum is over all spikes in the pre-synaptic neuron, *t*_*i*_ are the spike times and *δ* is the Dirac *δ* distribution as before. The weights are *w*_PNiKC_ = (6.75 + 0.844 $v$) nS, where $v \sim N\mathrm{(0,1)}$ is a normally distributed random variable, and *w*_eKCeKC_ = 50.6 nS. Synapses between iKCs and eKCs additionally follow a spike timing dependent plasticity (STDP) rule. At each spike occurrence,

8 $$\Delta w=\{\begin{array}{cc}A{e}^{-\frac{\Delta t}{{\tau }_{l}}} & \Delta t > 0\\ -A{e}^{\frac{\Delta t}{{\tau }_{l}}} & {\rm{otherwise}}\end{array}$$

9 $${w}_{ij}\mapsto {w}_{ij}+\Delta w$$

where *w*_*ij*_ symbolises the weight of a synapse between the spiking neuron and every other neuron it is connected to, and *w*_*ij*_ is clipped to the interval [0,*w*_max_] after Δ*w* is added. Δ*t* = *t*_post_ − *t*_pre_ is the time difference between pre- and post-synaptic spikes and we have adopted an all-to-all pairing rule, i.e. at each post-synaptic spike, all previous pre-synaptic spikes are considered and *vice versa*. The learning amplitude is *A* = *k*·0.1 nS, the STDP time scale *τ*_*l*_ = 10 ms, and *w*_max_ = *k* · 3.75 nS. The model was originally developed for 2,500 iKCs, which approximates the size of a *Drosophila* mushroom body, and we use the constant *k* = 2,500/*N*_iKCeKC_ as a scaling factor for parameters relating to the synaptic conductance from iKCs to eKCs. If *k* < 1, we set it to 1. Here, *N*_iKCeKC_ is the expected number of synapses to each eKC, i.e. *N*_*iKCeKC*_ = *N*_iKC_ if *N*_iKC_ < 10,000 and *N*_iKCeKC_ = 10,000 otherwise. This scaling avoids biologically unrealistic, too large inputs to eKCs.

The weights of the plastic synapses were initialized in two steps. First, all synapses were set to a low “inactive” weight *k*·*ω*·*w*_max_/10. Then, each weight was set to a higher, “active” weight *k*(2.5 + 0.5*ν*) with probability 0.2. Here $\omega  \sim U\mathrm{([0,1])}$ is again a uniform random variable in [0.1] and $\nu  \sim N\mathrm{(0,1)}$ a normally distributed random variable.

The PN neurons in the input layer are described by a spike source array, i.e. they emit spikes at pre-determined times and are otherwise not modeled in detail. We use a structured set of random input patterns. First we choose 10 basis input patterns by randomly choosing 20 active PNs. Each of these input patterns is multiplied into *N*_rep_ variants by changing the identity of each of the active PNs with probability 0.1. The number *N*_rep_ is determined such that the overall runtime is as desired (see below) and all variants are presented once. Patterns are presented every 50 ms with a random jitter chosen uniformly between 0 and 2 ms. All other neurons are described by Hodgkin-Huxley equations as in the COBAHH model above, Eq. (4) and Table 3, but parameterised slightly differently with *C*_*M*_ = 0.3 nF, *g*_*L*_ = 26.7 nS, *g*_Na_ = 7.15 μS, *g*_K_ = 1.43 μS, *V*_*L*_ = −63.56 mV, *V*_Na_ = 50 mV, and *V*_K_ = −95 mV. All Hodgkin-Huxley neurons were initialised with *V* = *V*_*L*_, *m* = 0, and *h* = 0.5.

The source code of the two model networks is published alongside the entire benchmarking code and results at https://github.com/brian-team/brian2genn_benchmarks.

### Benchmarks

Benchmarks were run on a number of different workstations, with different GPUs installed ranging from a standard consumer card (Quadro K2200) to a more powerful gaming GPU (TITAN Xp), an older computing accelerator model (Tesla K40c) to the most recent and most powerful accelerator (Tesla V100). The different configurations for benchmarking are listed in Table 1. We used Brian 2, version 2.2^25^, GeNN version 3.2^26^, and Brian2GeNN version 1.2^27^ for our benchmarks.

In initial benchmarks we tested the models when run with “monitors”, Brian’s mechanism for recording the activity during a simulation, and without. We observed that when monitoring the state of a few neurons, monitors play virtually no role in the context of the two models used as benchmarks here. We, therefore, only present the benchmarking figures without monitors in place. For the runs using Brian2GeNN, we used GeNN’s Yale sparse matrix representation^9^ throughout. While for smaller models, dense matrix representations may have speed advantages, the more relevant mid- and large-scale models would lead to “out of memory” failure on all tested GPUs with either of GeNN’s dense matrix representations. Even with sparse matrix representation, some of the largest simulation sizes could not be run on all GPU models because of the difference in size of the GPU memory on the employed devices. The corresponding data points were omitted from the benchmark figures.

## Data Availability

Brian2GeNN is developed publicly on github (https://github.com/brianteam/brian2genn). The scripts and raw results of the benchmark runs are available at https://github.com/brian-team/brian2genn_benchmarks.
